# Healthy worker survival effect at a high-altitude mine: prospective cohort observation

**DOI:** 10.1038/s41598-022-18331-4

**Published:** 2022-08-16

**Authors:** Denis Vinnikov, Viktor Krasotski

**Affiliations:** 1 al-Farabi Kazakh National University, 71 al-Farabi avenue, Almaty, 050040 Kazakhstan; 2 Peoples’ Friendship University of Russia (RUDN University), Moscow, Russian Federation; 3 Kumtor Gold Company, Bishkek, Kyrgyzstan

**Keywords:** Diseases, Risk factors

## Abstract

Very little is known about the factors of healthy worker survival effect at high-altitude mines. We conducted this cohort observation of the new hires for a high-altitude gold mine in Kyrgyzstan with the aim to ascertain predictors of survival at work. All new hires in 2009 through 2012 for a high-altitude gold mine (3600–4500 m above sea level) were followed up to January 2022. We tested the association of demographic, physiological predictors and diagnoses at the pre-employment screening with non-survival at work in Cox proportional hazards yielding hazard ratios (HR) with their 95% confidence intervals (CI). The cumulative observation time was 5190 person-years. Blood pressure at pre-employment, lung function, the diagnoses of essential hypertension, chronic obstructive pulmonary disease (COPD) or any other analyzed physiological variables were not associated with non-survival at work. However, smoking (HR 1.55; 95% CI 1.10; 2.17) increased the likelihood of non-survival at work, independent of any diagnosis or lowland residence (HR 1.95; 95% CI 1.31; 2.90). Adjusted for covariates and all diagnoses, having chronic rheumatic fever (HR 10.95; 95% CI 2.92; 33.92), hemorrhoids (HR 1.32; 95% CI 1.01; 3.75), adhesive otitis (HR 1.74; 95% CI 1.05; 2.89) or obesity (HR 1.71; 95% CI 1.01; 2.88) were associated with non-survival at work with time. This prospective observation of new hires for a high-altitude mining operation demonstrated that selected diagnoses, smoking and lowland residence elevated the risk of early exit in prospective workers.

## Introduction

Work at high-altitude gold mines exposes employees not only to a wide range of occupational hazards, such as respirable dust, cyanides, explosion gases and shift work, but to a specific hypobaric environment in a chronic intermittent pattern. Two or three weeks in hypobaric hypoxic conditions shift with normoxia, putting cardiovascular, respiratory and endocrine compensatory mechanisms in chronic stress. At present, much is known about physiological adaptive response to hypobaric hypoxia in high-altitude miners^1–5^, but determinants of long-term uncomplicated employment at the mines remain very poorly understood. Longitudinal observations studies in these specific occupational cohorts are hard to accomplish, because these few mines in the world are remote and predominantly located in developing countries with poor coordination with research institutions.

Because employment at high-altitude mines is associated with elevated health risks^6^ fitness to work is usually a subject for governmental regulatory control over the medical institutions issuing clearance for work. In Kyrgyzstan and neighboring countries, full medical examination procedure is regulated by the Orders or Regulations of the Ministries of Health, whereas the current regulations are basically a mild transformation of older documents, when little or no evidence was available as to whether prospective workers with selected medical conditions should or should not be allowed to work. At a time when these regulations were issued, almost no prospective observational studies were published assessing the clinical prognosis and the probability of deterioration of medical conditions and diseases during employment. Many medical contraindications to work were based on the known worsening prognosis in a conventional medical practice of the general population at low altitude, but many were arbitrary.

Examples of the so-called absolute contraindications for high-altitude employment at the mines in Kyrgyzstan are chronic nervous diseases, obliterating endarteritis, severe varicose veins, hemorrhoids with frequent exacerbations and bleeding, hernias with a tendency to strangulation, peptic ulcer disease, skin diseases with frequent exacerbations, bronchial asthma, pulmonary tuberculosis and other. Timely and accurate diagnosis at a time of pre-employment screening is critical, because the panel of doctors has to make a decision sooner and with no errors in diagnoses, as this will preclude further lawsuits of an employee, should he or she be made fit but further medical complications or death occur at site. Some of these current contraindications may not have a firm evidence from the literature that they will deteriorate in workers at high altitude. Therefore, high-quality prospective observations may shed more light on whether clinical prognosis of initially non-severe conditions will worsen at altitude.

Furthermore, healthy worker survival effect, widely prevalent in may occupations, may also play role in workers’ selection with time at such mines, but little is known about the predictors of survival at work. Cigarette smoking may be one of these predictors, because it has been shown to increase the likelihood of acute mountain sickness in workers^7^. The contribution of obesity as another example has also have to be elucidated, but no studies confirming this to-date have been published. We, therefore, planned and conducted this cohort observation of the new hires for a high-altitude gold mine in Kyrgyzstan with the aim to ascertain predictors of survival at work.

## Results

### Baseline characteristics of the cohort

The cumulative observation time was 5190 person-years. At inclusion, this was a middle-aged group of workers (median age 34 years) with high smoking prevalence (44%) and mostly middle-altitude residents (the altitude of terrains around Issykul lake 1600 m above sea level (MASL)), whom we grouped into three occupational groups. These were non-production employees (12%); production but not heavy-duty vehicle operators (29%); and heavy-duty vehicle operators (59%). Only 8% of new hires were obese with blood pressure and heart rate within the normal limits (Table 1). Up to 23% of examined subjects showed blood hemoglobin level exceeding 23% because of some high-altitude exposure before. Lower cigarette smoking prevalence, systolic, but not diastolic BP and blood hemoglobin were found in female workers compared to men. During the first ascent to the mine, the median SpO_2_ was 89%.

**Table 1:** The overall and sex-specific demographic and physiological characteristics of the cohort at entry.

Characteristics	All (n = 569)	Males (n = 541)	Females (n = 28)
**Demographics and job demands**			
Lowland residents, n (%)	88 (15)	81 (15)	7 (25)
Age at entry, years, median (IQR)	34 (28; 40)	34 (28; 40)	33.5 (28; 39)
Smoking Status			
Current cigarette smoking, n (%)	253 (44%)	252 (47)	1 (4)*
Cigarettes per day, median (25th–75th percentile) for smokers	8 (5; 10)	8 (5; 10)	5 (5; 5)*
**Physiological variables**			
FEV_1_/FVC %, median (IQR)	80 (76; 85)	80 (76; 85)	83 (76; 87)
FEV_1_/FVC < 0.70, n (%)	34 (6%)	32 (6)	2 (7)
BMI, kg/m^2^, median (IQR)	24.2 (22; 27.1)	24.2 (22; 27.2)	24 (21; 27)
Obese, n (%)	46 (8%)	45 (8)	1 (4)
BP systolic, mmHg, median (IQR)	122 (120; 130)	125 (120; 130)	120 (106; 123.5)*
BP diastolic, mmHg, median (IQR)	80 (70; 80)	80 (70; 80)	70 (70; 80)
Heart rate, beats per minute, median (IQR)	70 (66; 78)	70 (66; 78)	70 (60.5; 80)
Hemoglobin, g/l, median (IQR)	170 (160; 180)	171 (161; 180)	132 (123; 155)*
Hemoglobin > 180 g/l, n (%)	130 (23%)	130 (24)	0 (0)*
Erythrocyte sedimentation rate, mm/h	4 (2; 7)	4 (2; 6)	10 (6; 13)
SaO_2_ at first ascent, %, median (IQR)	89 (87; 91)	89 (87; 91)	90 (89; 91)*

*IQR* interquartile range, *FEV*_*1*_ forced expiratory flow in 1 s, *FVC* forced vital capacity, *BMI* body mass index, *BP* blood pressure, *SaO*_*2*_ blood oxygen saturation. Lowland is defined by residence at less than 1000 m above sea level. *p < 0.05.

### The overall picture of survival at work

The observation was stopped by the end of December 2021; thus, resulting in 13 years of follow-up for those hired in 2009; 12 years for those hired in 2010; and the corresponding follow-up duration for 2011 and 2012 hires were 11 and 10 years [median time of observation 119 (IQR 102; 139) months]. One-hundred and fifty-five study participants (cumulative incidence 27%) of the initially recruited cohort of 569 workers were reported as failed completion for a medical, non-medical reason or death. In those who left the company prematurely, employment duration ranged from 6 to 154 (median 48; IQR 36; 86) months. Ninety-five percent of those who did not survive until the end of observation and exited the study were men (148 of 155 workers). Nine employees died, of whom one case occurred at mine site as a result of a fatal accident, four workers died at home with no autopsy leaving the reason for death unstated, one more died from heart attack, two more from cancer and the remaining ninth subject died at home from alcohol intoxication.

### Diagnoses at the annual screening and univariate analyses

As Table 2 shows, the most prevalent diagnosis was deviated septum, found in almost every fifth employed worker, followed by myopia or astigmatism or both (17.0%) and elevated serum lipids (15.6%). Tooth caries and adhesive otitis were also among the most frequently found conditions with a prevalence exceeding 10%. Diseases with very low prevalence, such as arrythmia, mesotympanitis, cataract, gall bladder stones, thyroid diseases, varicocele, gastroesophageal reflux disease (GERD) and strabismus were not included in the analysis. None of the diagnoses except rheumatic fever were associated with survival at site effect in the univariate comparisons. However, there were significantly more lowland residents and smokers (52 vs. 42%) in those who left work prior to the observation completion. We found no difference in the occupational and sex structure between survivors and non-survivors. Baseline physiological variables, such as blood pressure, lung function, hemoglobin and erythrocyte sedimentation rate recorded at the study entry did not affect survival either. Oxygen saturation measured at first ascent during the routine medical examination at mine site did not differ between two groups either.

**Table 2:** Predictors of successful survival at work in the univariate comparisons.

Characteristics	All (n = 569)	Survivors (n = 414)	Non-survivors (n = 155)
Males, n (%)	541 (95)	393 (95)	148 (95)
Lowland residents, n (%)	88 (15)	53 (13)	35 (23)*
Current cigarette smoking, n (%)	253 (44%)	173 (42)	80 (52)*
**Occupational groups**			
Non-production	69 (12)	48 (12)	21 (14)
Production, but not vehicle operators	167 (29)	125 (30)	42 (27)
Mine truck and other heavy-duty vehicle drivers	333 (59)	241 (58)	92 (59)
BP systolic, mmHg, median (IQR)	122 (120; 130)	120 (120; 130)	125 (120; 130)
BP diastolic, mmHg, median (IQR)	80 (70; 80)	80 (70; 80)	80 (70; 80)
Heart rate, beats per minute, median (IQR)	70 (66; 78)	70 (64; 78)	70 (66; 78)
Hemoglobin, g/l, median (IQR)	170 (160; 180)	170 (160; 180)	171 (160; 181)
Erythrocyte sedimentation rate, mm/h	4 (2; 7)	4 (2; 6)	4 (2; 7)
**Diagnoses at entry, n (%)**			
Deviated septum	106 (18.6)	73 (17.6)	33 (21.3)
Myopia/astigmatism	97 (17.0)	63 (15.2)	34 (21.9)
Elevated cholesterol or triglycerides	89 (15.6)	70 (16.9)	19 (12.3)
Caries	68 (12.0)	48 (11.6)	20 (12.9)
Adhesive otitis	59 (10.4)	37 (8.9)	22 (14.2)
Mycotic disease of nails or skin	49 (8.6)	34 (8.2)	15 (9.7)
Obesity	46 (8.1)	28 (6.8)	18 (11.6)
Sensorineural deafness	45 (7.9)	31 (7.5)	14 (9.0)
FEV_1_/FVC < 70%	34 (6.0)	23 (5.6)	11 (7.1)
Hemorrhoids	30 (5.3)	19 (4.6)	11 (7.1)
Kidney stone disease	27 (4.7)	20 (4.8)	7 (4.5)
Elevated fasting glucose	16 (2.8)	10 (2.4)	6 (3.9)
Conductive deafness	16 (2.8)	13 (3.1)	3 (1.9)
Essential hypertension	15 (2.6)	19 (4.6)	6 (3.9)
Pterygium	15 (2.6)	10 (2.4)	5 (3.2)
Dichromacy/trichromacy	12 (2.1)	8 (1.9)	4 (2.6)
Varicose veins	12 (2.1)	9 (2.2)	3 (1.9)
Chronic gastritis	10 (1.8)	8 (1.9)	2 (1.3)
Chronic pyelonephritis	9 (1.6)	6 (1.4)	3 (1.9)
Chronic back pain	8 (1.4)	6 (1.4)	2 (1.3)
Chronic viral hepatitis	7 (1.2)	6 (1.4)	1 (0.6)
Alcohol addiction	7 (1.2)	4 (1.0)	3 (1.9)
Psoriasis	7 (1.2)	4 (1.0)	3 (1.9)
Chronic neck pain or headache	7 (1.2)	5 (1.2)	2 (1.3)
Scoliosis	6 (1.0)	5 (1.2)	1 (0.6)
Hemoglobin < 120 g/l	5 (0.9)	5 (1.2)	0 (0)
Chronic rheumatic fever*	4 (0.7)	1 (0.2)	3 (1.9)

*IQR* interquartile range, *FEV*_*1*_ forced expiratory flow in 1 s, *FVC* forced vital capacity, *BP* blood pressure. Lowland is defined by residence at less than 1000 m above sea level. *p < 0.05.

### Multivariate survival analysis

**Figure 1:**
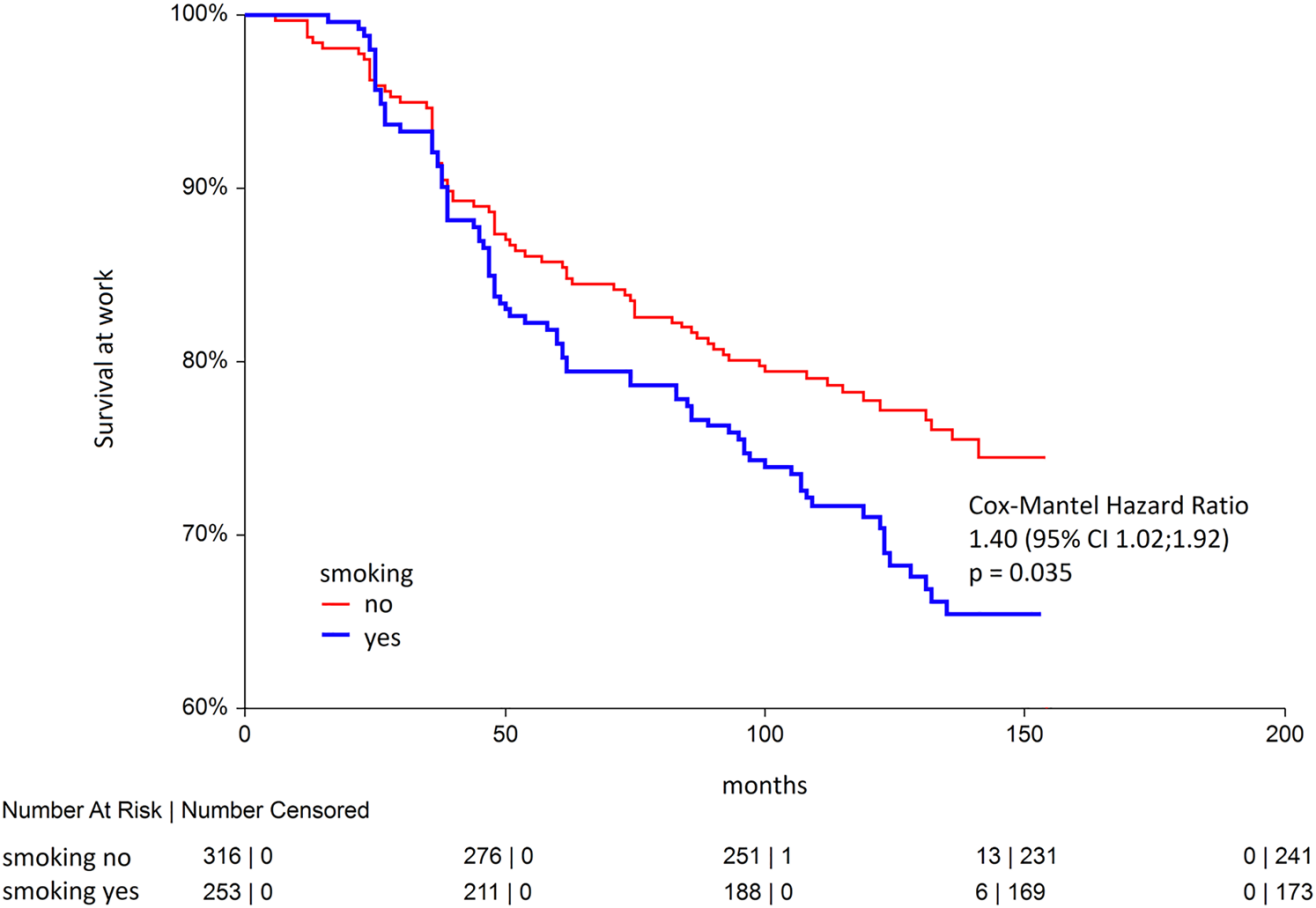
Kaplan–Meier curves for smokers and non-smokers in a prospective observation.

**Figure 2:**
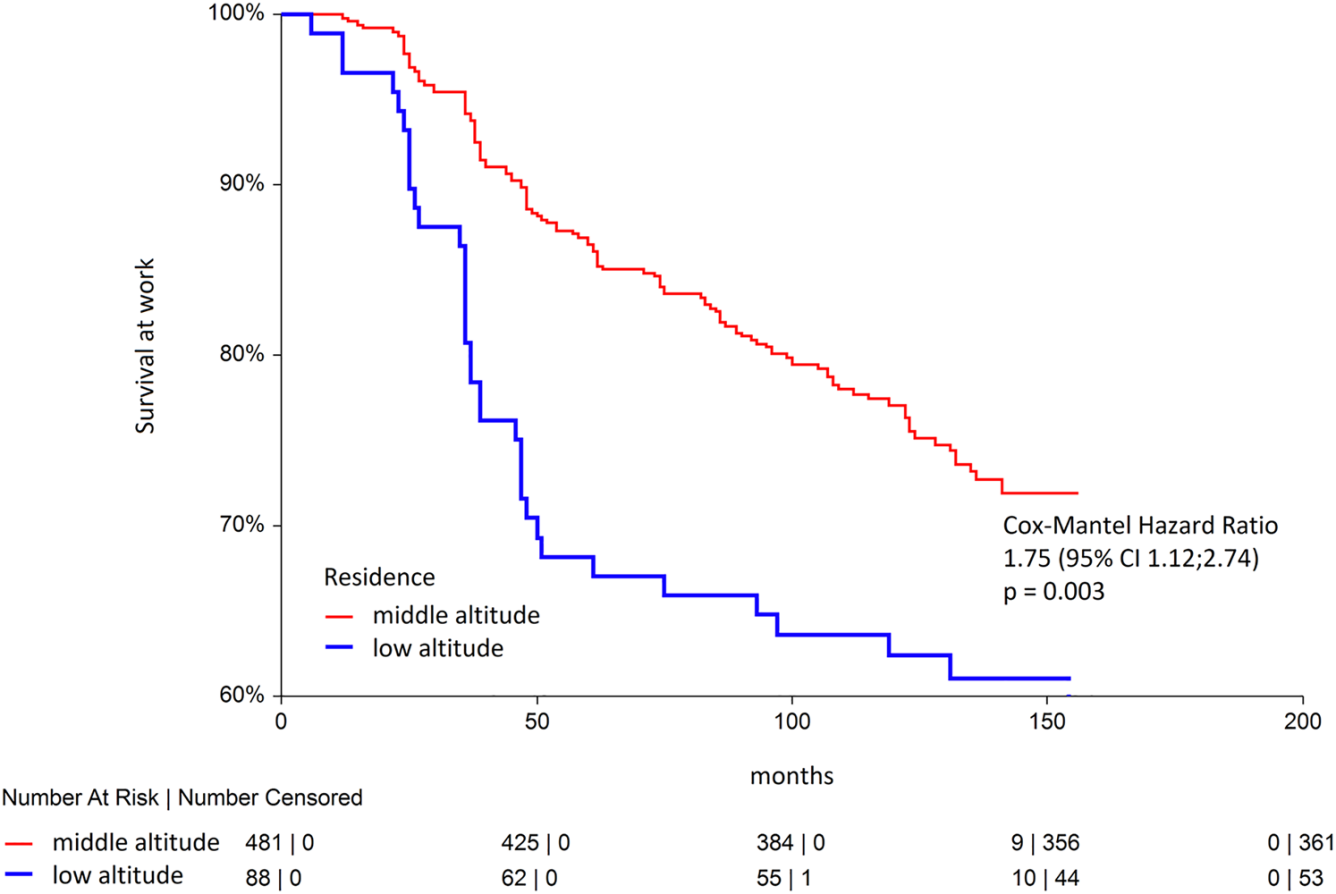
Kaplan–Meier curves for lowland and middle-altitude residents in a prospective observation.

With elapsing time at work, there were more smokers leaving work at high altitude (Fig. 1), and the probability of employment discontinuation in smokers was 1.4-times of that in non-smokers (HR 1.40; 95% CI 1.02; 1.92). We also found that being a low-land resident was also a strong predictor of non-survival at work at the mine (Fig. 2), whereas the effect was even stronger than that for smoking (HR 1.75; 95% CI 1.12; 2.74). We then tested these significant predictors of non-survival at work together with CRD in one model (Model 1) and then adjusted for all other diagnoses in another model (Model 2). In such regression models, the effects of both smoking, living at low altitude and CRD were even stronger than in univariate comparisons, HR 1.55; 95% CI 1.10; 2.17 and HR 1.95; 95% CI 1.31; 2.90, accordingly (Table 3). Despite relative few cases of CRD in miners who were hired, the likelihood of leaving work was almost tenfold greater compared to subjects with no such condition, and the effect was highly significant (HR 9.95; 95% CI 2.92; 33.92). Finally, when all diagnoses were tested in the adjusted analysis, hemorrhoids (HR 1.95; 95% CI 1.01; 3.75) and adhesive otitis (HR 1.75; 95% CI 1.05; 2.89) increased the odds of non-survival at high-altitude mining work with time.

**Table 3:** Cox proportional hazards for selected predictors of non-survival at high-altitude work in crude and adjusted analyses.

	Univariate		Model 1		Model 2	
	HR	p	HR	p	HR	p
Residence at low altitude	1.76	< 0.01	1.86	< 0.01	1.95	< 0.001
Cigarette smoking	1.40	< 0.05	1.49	< 0.05	1.55	< 0.05
CRD	6.93	< 0.001	8.92	< 0.001	9.95	< 0.001
Obesity		NS	–	–	1.71	< 0.05
Adhesive otitis		NS	–	–	1.74	< 0.05
Hemorrhoids		NS	–	–	1.95	< 0.05

*CRD* chronic rheumatic disease, *HR* hazard ratio, *Model 1* adjusted for each other (residence; smoking and CRD) , *Model 2* adjusted for variables in Model 1 and all diagnoses (N = 27).

## Discussion

To our best knowledge, this is the first presentation in the world literature providing a comprehensive portrait of healthy worker survival effect at the high-altitude gold mine in a prospective cohort observation among newly hired personnel of all occupational groups. We also assessed how individual diagnoses at pre-employment screening in subjects cleared to work affected their survival at work with time. We found that none of included physiological variables or occupations were associated with survival, but being a low-land resident and a smoker were. These two were independently associated with employment discontinuation with time, as did chronic rheumatic fever with even greater effect and hemorrhoids with adhesive otitis with the effect somewhat comparable to being a lowland resident and a smoker.

Review of the relevant scientific literature shows that there exist only sporadic reports of the cohort observational studies of high-altitude mining personnel^8^, and none of them were designed to ascertain the survival or the way healthy worker effect worked in this occupational group. Most of such longitudinal studies focused on cardiovascular outcomes with time in those exposed to intermittent hypoxia for work. One of these studies examined the association of work at altitude with the incidence of cardiac abnormalities and found that 60% of 173 initially hired workers had developed cardiac abnormalities, predominantly right heart enlargement and left ventricular diastolic dysfunction^9,10^. An earlier systematic review confirmed such effect, but concluded confounding from socioeconomic status and obesity^11^. However, our current cohort after 1 year of follow-up demonstrated no blood pressure elevation when working at altitude 3600–4500 m above sea level^12^. Other published studies are limited to very short observation periods, usually within days since exposure initiation, or to short overall duration of construction activities or enterprise^6^.

Despite insufficient data on survival at work in the mining companies at high altitude, pre-employment screening panel of doctors have to make critical decision on whether placing a subject in the workplace at altitude will not put him at high risk of deterioration. Robust medical recommendations exist with regard to all chronic medical conditions in the advanced or terminal stage and high risk of decompensation, such as coronary artery disease, despite no clinical worsening at ascent in the short run^13^. Because miners go to high altitude for years of work, associated with some physical workload, sometimes graded as heavy, making coronary artery disease a contraindication is pertinent and sensible, as also shown elsewhere^6^. In our study, subjects with all such conditions were made unfit to work, thus, limiting the circle of diagnoses under study in a prospective observation to relatively mild conditions. Therefore, natural observation over severe medical conditions, including coronary artery disease, epilepsy or decompensated diabetes as a few examples to test the prognosis at work at high altitude mines is almost not feasible. In case of emergency, these patients will have lower chances to survive due to remoteness from the centers of higher-level care.

On the contrary, contraindications with diagnoses, which have been shown to improve at high altitude and subject to high-altitude climate therapy, such as bronchial asthma, should be revisited. High-altitude climate has been used for decades to treat asthma and maintain control with lower doses of steroids; furthermore, meta-analysis showed that exposure to high altitude would increase lung function^14^. Precise mechanism of high-altitude positive effect has yet to be fully understood^15,16^; however, allergen avoidance^17^ and elevation in endogenous cortisol^18^ and the overall inflammation suppression^19,20^ are believed to make a major contribution to this. Therefore, making prospective workers with controlled asthma on treatment unfit to work should be questioned, if no exposure to allergens and irritants is expected in the workplace and controlled asthma on treatment is documented. No prospective studies, however, exist, demonstrating that work at high altitude is safe for such subjects, and should be initiated in future.

The current medical regulations in Kyrgyzstan prohibit work at high altitude an in the remote setting for patients with uncontrolled hemorrhoids with frequent exacerbations or bleeding. We, however, did not find any preceding studies on the prognosis of this diagnosis at high altitude. Some of our patients with mild disease were fit to work; however, our new data from this finding should alert the screening panel of doctors whether a patient will survive at altitude even with a mild disease. Another medical diagnosis with a strong association with early leave in the current report was chronic rheumatic fever. Because currently the disease has very low prevalence as a result of widespread highly potent antibiotic use and almost all cases do not have signs of acute inflammation, but a compensated valvular defect only, these patients have obtained clearance and have been eventually employed. We could not identify reports on the clinical prognosis of this diagnosis in high-altitude workers, but presentation of rheumatic fever clinical attributes at high altitude were published in the last century^21,22^. A ten-fold increase in the likelihood of early work discontinuation in these patients in the current study, even if the disease does not exhibit the signs of acute inflammation, necessitates a firm negative decision on fitness of such patients at pre-employment screening.

Long before this study, cigarette smoking was known to be associated with a wide range of negative health outcomes in general and in the occupational setting. Accelerated lung function decline, elevated risk of acute mountain sickness^7^ were those few among negative health effects of smoking in the working population at high altitude. Consistent with another study of the association of smoking with non-survival at work^23^, we now supplement this notation with a similar effect at the high-altitude mine. Furthermore, in a large 30-year cohort observation of veterans, they demonstrated that smoking-related diseases contributed most in mortality of those who left prematurely^24^. Taken together, we conclude that smoking is an important modifiable risk factor for early work leave; therefore, persistent advice on smoking cessation for mine employees at high altitude should be supported by the management and not ignored by the medical professionals.

We hypothesized that body mass index played role in the overall adaptation to high altitude. Overall lower prevalence of obesity at high altitude has been demonstrated in many prior studies, and the underlying mechanism is likely a combination of greater energy expenditure with other factors^25,26^; therefore, travel to high-altitude terrains has been proposed as an obesity management program^26^. Nevertheless, given that obesity is strongly associated with obstructive sleep apnea^27,28^, its combination with hypoxia-induced periodic breathing and desaturation at high altitude may be much more pronounced in subjected with high BMI and obesity. Furthermore, in a recent study of a large sample of short-term visitors, they found greater desaturation at the altitude in subjects with greater BMI^29^. We, therefore, hypothesized that obese workers would have had increased odds of premature leave, and our findings confirmed such association.

The strengths of this study include the pioneer long-term cohort observation of survival at work; long period of observation; diagnoses verification by a panel of qualified narrow specialists; and detailed physiological, demographic and personal medical history analysis as risk factors for survival. Inclusion of only one high-altitude mining company, though the largest gold producer in the whole region of Central Asia is a limitation of our analysis. We also consider inability to track further vital status of those who did not survive at work and left the company as another limitation. Furthermore, we had no access to detailed medical information of deceased patients at home, when causes of death remained unknown. In addition, we could not test predictors specific for high-altitude environment, and those tested were also present in low-altitude occupational groups. Therefore, our findings should be interpreted and applied for fitness to work with caution. Finally, we did not access indicators of socioeconomic status, which we believed could confound survival at work, but given that all employees were all from one company with similar pay rates, dramatic difference in the socioeconomic status was unlikely.

In conclusion, this first observational cohort study of new hires for a high-altitude gold mining company demonstrated that physiological variables at pre-employment screening were poor predictors of successful survival at work. Being a smoker and living at low versus middle altitude for new hires were independent predictors of non-survival at work. Some association of chronic medical conditions with non-survival has also been confirmed providing background and more evidence to correct or improve medical regulations for screening in future.

## Materials and methods

### Study design

This prospective cohort observational study of all newly hired personnel of the leading gold mining company in Kyrgyzstan was approved by the Committee on Bioethics of Kyrgyz State Medical Academy. All methods were performed in accordance with the relevant guidelines and regulations, and informed consent was obtained from all subjects. Details on the cohort construction, eligibility criteria and occupational structure were published earlier in the paper demonstrating the risk of acute mountain sickness from the first year of prospective observation^30^. In brief, all newly hired personnel who underwent pre-employment medical screening, as mandated by the local Order 70 of 2000 of the Ministry of Health, to work at mine site, were considered eligible for inclusion. Mine site is located at an elevation of 3600–4500 m above sea level in Tien Shan mountains, in the Issykul Province of Kyrgyzstan, 600 km away by road from Bishkek. Open-pit mine along with the mill and living camp are situated next to each other. Workers commute to mine site by bus either from Issykul Lake (altitude 1600 m above sea level) or from Bishkek (700 m above sea level). Work shifts usually last for 2 weeks at site with 12-h workdays, followed by subsequent 2 weeks of rest at home. Such exposure pattern, referred to as ‘high-altitude exposure’ reflects the one of ‘chronic intermittent hypoxia’^31^, and these terms are used interchangeably in this presentation.

### Medical examination

Medical examination at entry is a 2-day procedure comprising complete blood cell count with erythrocyte sedimentation rate; blood biochemistry, which includes glucose (and even glucose tolerance test and glycoHHB in case of abnormality), lipid profile, urea, creatinine, alanine aminotransferase, aspartate aminotransferase, gamma-glutamiltranspeptidase, and other metabolites upon indications; 12-lead electrocardiography; frontal chest X-ray; air conductivity audiometry and spirometry with bronchodilation. Additionally, vision acuity and intraocular pressure are measured at the ophthalmologist’s examination, whereas bone conductivity hearing test may be accomplished at the ENT visit upon indications. Prospective employees may be asked to pass other ancillary tests such as echocardiography, stress-test, coronary angiography, gastroscopy, ultrasonography, should the panel doctor consider them needed for the diagnosis. Finally, subjects are then seen by a panel of eight, later nine specialists, including internal diseases doctor, dermatologist, gynecologist, ENT, ophthalmologist, surgeon, psychiatrist, neurologist and cardiologist.

Smoking status is routinely collected with a questionnaire, categorizing subjects to never-, former and current cigarette smokers. Average number of cigarettes smoked a day with years of smoking are also recorded, followed by exhaled carbon monoxide concentration measurement as a way of smoking intensity verification with portable PiCO Smokerlyzer (Bedfont, UK). Other tests and examinations, including lung function testing were described before^30^. During the entire observation period, we recorded all events of work leave with the corresponding reasons for leave coded in the medical database. These data were additionally matched with the human resources data on the duration of work contract.

### Statistical analysis

All prospective employees who successfully completed medical examination, commenced their work at mine site and worked for at least one year were included in this analysis and were treated as at risk for non-survival at work by the end of observation. As in the preceding report from this cohort assessing the risk of acute mountain sickness^30^, we screened the database on all events coded as death, resignation or permanent work discontinuation in the company, when observations were censored. Alternatively, we censored these in January 2022, when the study was completed, if none of the leave records existed. Cox proportional hazards regression models were constructed to assess the risk of leave associated with selected predictors, including demographics, smoking status, basic physiological variables from the pre-employment screening and the first ascent to the mine, and diagnoses from the first screening.

At first, data were screened for normality, and since most variables were non-normally distributed, we used non-parametric tests for univariate comparisons in this analysis, such as Mann–Whitney U-test. Such univariate comparisons also included testing all selected predictors between survivors by the end of observation and non-survivors for various reasons. Cigarette smoking and residence at low altitude were expressed as binary variables (yes/no). Most common twenty-seven diagnoses were also included in such comparisons, whereas diseases with very low prevalence were ignored. In the crude models, we calculated hazard ratios (HR) of the variable of interest for non-survival (premature leave for any reason) with their corresponding 95% confidence intervals (CI). Of note, patients with any diagnoses from the list of mandatory contraindications to work at high altitude were made unfit to work and thus could not commence employment. Therefore, tested diagnoses in this cohort observation only included non-severe conditions. We then adjusted significant predictors from the univariate regression models for each other in an adjusted model (Model 1). This model included such variables as residence at low altitude, cigarette smoking and chronic rheumatic disease. Furthermore, we then constructed Model 2 with all variables from Model 1 and all 27 diagnoses, thus, adjusting for each other. The overall number of tested variables in Model 2 was 30. All tests were completed in NCSS 2021 (Utah, USA).

## Data Availability

All data generated or analysed during this study are included in this published article.
